# Editorial: Hirschsprung disease: genetic susceptibility, disease mechanisms and innovative management in the multi-omics era

**DOI:** 10.3389/fped.2023.1274735

**Published:** 2023-08-31

**Authors:** Consolato M. Sergi, Josef Hager

**Affiliations:** ^1^ Anatomic Pathology Division, Children’s Hospital of Eastern Ontario (CHEO), University of Ottawa, Ottawa, ON, Canada; ^2^ Department of Laboratory Medicine and Pathology, University of Alberta, Edmonton, AB, Canada; ^3^ Pediatric Surgery, University Clinic of Surgery, Medical University, Innsbruck, Austria

**Keywords:** aganglionic diseases, hirschsprung, hirschsprung associated enterocolitis, hirschsprung allied disorders, gut, children

**Editorial on the Research Topic** Hirschsprung disease: genetic susceptibility, disease mechanisms and innovative management in the multi-omics era

**Figure 1:**
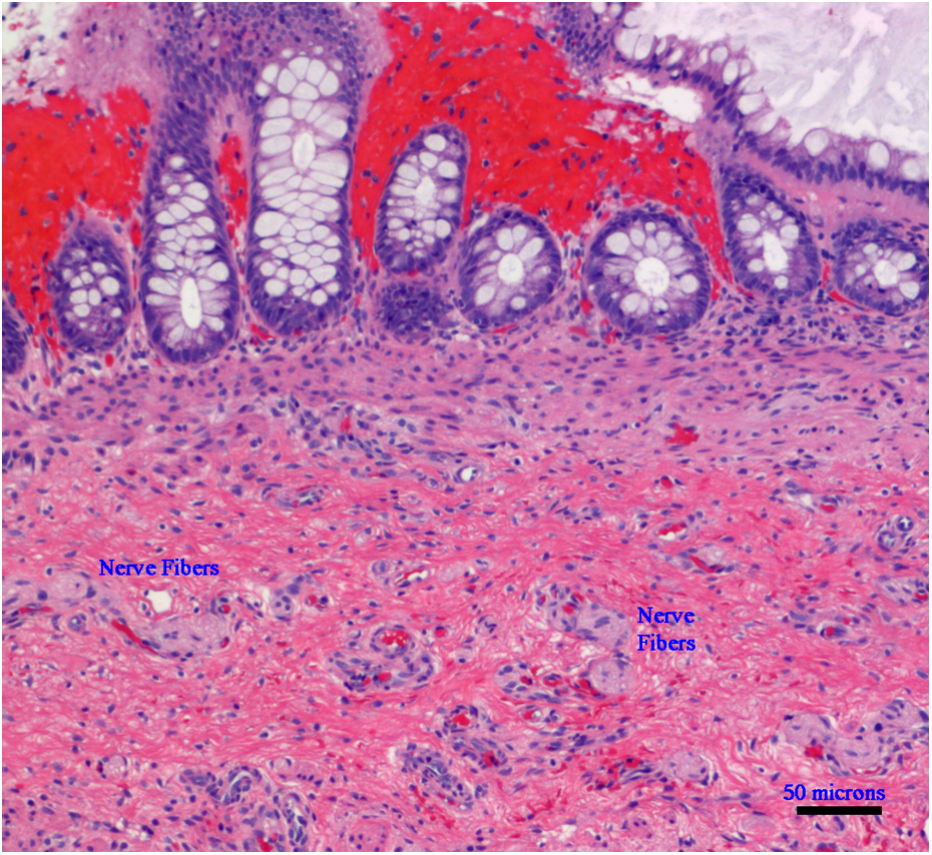
Microphotograph of a rectal suction biopsy showing no ganglion cells, but exclusively nerve fibers (Hematoxylin and Eosin staining, ×100, scale bar: 50 micrometers).

Hirschsprung disease (HSCR) is a puzzling subject for researchers and the story of its etiology as well as non-surgical cures have been the subject of extensive ongoing research (1). HSCR is a heterogeneous disease with an incidence of 1.5–2.8 per 10,000 births. It is characterized by varying lengths of cells, a lack of ganglion cells, and hypertrophy of nerve fibers, and has a preponderance among male patients (M:F-5:1), with a variable familial incidence and occasional syndromic involvement (2, 3). In the neonatal period, about 80% of infants (∼10% of whom are preterm) present with a delayed passage of meconium (beyond 24 h), an increasingly distended abdomen, and intermittent vomiting. In individual cases, around 12% of these children may already have an impaired general condition caused by pre-existing or developing enterocolitis. In about 10% of children with short-term HSCR, symptoms only appear later in life, usually during the switch from breastfeeding to pap food, mainly since the soft breast milk stool can be transported through the non-innervated sigmoid/rectal segment without any problems. These children are characterized by an intermittent refusal to eat and rapidly increasing constipation or encopresis. After clinical, radiological, and manometric investigations, the current standardized method to diagnose HSCR is a rectal biopsy above the dentate line with the help of a suction device with the subsequent histologic examination (4) (Figure 1).

HSCR is a disease that significantly affects patients and families, and the current cure is exclusively surgical. The promise of the Omic-Era is encouraging because research is being undertaken to identify niches in which researchers and physicians can collaborate and improve the outcomes for patients affected by this terrible disease (2). The era of high-throughput technologies has the impetus to accelerate its scale dramatically in the 21st century. Genomics, epigenomics, transcriptomics, proteomics, metabolomics, glycomics, and lipidomics offer an outstanding opportunity for holistic investigation and contextual understanding of the pathogenesis of gastrointestinal disease for precise diagnosis and tailored treatment. This Research Topic aimed to stimulate the field, inviting researchers to contribute to this collection and further knowledge of this disease.

Fan et al. report the unusual finding of an anorectal malformation (ARM) with HSCR (Fan et al.). ARM and HSCR are rarely reported together. In this study, the authors report an overall incidence of 2.4%–3.4% of cases. The paper is exciting and very precise, with spectacular images. Everything is explained in detail, but importantly, in the case of anal atresia, the simultaneous occurrence of HSCR should be considered.

The REarranged during Transfection (RET) is one of the most significant genes discovered by 24 genes associated with HSCR (Iskandar et al.). RET's somatic mosaicisms have been identified in HSCR but are poorly recognized. Many of the contributions to this special issue highlight the frequency of rs2435357somatic mutation of RET in HSCR patients. Iskandar et al. suggest that somatic mosaicism in HSCR patients is not uncommon, supporting the complexity of the pathogenesis of this gastrointestinal disorder (Iskandar et al.). They confirm that the RET rs2435357 is a paramount genetic risk factor for HSCR patients. With the help of molecular genetics, it is even possible to detect specific changes in the patient's genetic information. The authors rightly speak of a mosaic status. In this context, RET rs2435357 is of particular importance in HSCR patients. It would be interesting to identify the burden of each mutation in causing HSCR in the future.

Wang et al. used the robust linear model (RLM) statistical method. Robust regression uses an iterative approach to assign a weight to each data point. The RLM algorithm uses the least-squares approach to identify the curve that outbursts the data bulk. The final event is to minimize the effects of outliers. These authors used RLM for screening plasma human autoimmune antigen microarrays. They quantitatively assessed enzyme-linked immunosorbent assay (ELISA) values with single-stranded DNA (ssDNA) antibody levels. They found that ssDNA antibodies in HSCR plasma were considerably higher than those in healthy and disease controls. Furthermore, ssDNA antibodies differentiated HSCR from non-HSCR patients, accomplishing an area under the curve (AUC) of 0.917, harboring a sensitivity of 96.99% and a specificity of 74.63%. The considerations of this work are interesting, especially since they mean an extension of diagnostics. The occurrence of the ssDNA antibody could be promising, especially in premature babies.

We are still searching for the etiology of HSCR, and the findings of Ji et al. may help us understand one of the life-threatening conditions associated with HSCR. Neuroimmune instruction intercedes the incidence and progress of enteritis. Single-cell RNA sequencing has been critical in decrypting several intracellular processes and signaling mechanisms in the last few years. Both human and mouse gut nervous system (ENS) components demonstrate that healthy gut neuronal cells prompt mediators and cell surface molecules, which can interconnect with innate and adaptive immunologic pathways. The enhancement of the neuromodulatory effect may prevent this entity from occurring. Vasoactive intestinal peptide, substance P, and neuropeptide Y carry a cationic charge that can break the bacterial membranes and kill the microorganisms. The authors describe the characteristics of the interaction between intestinal nerve cells and immune cells. We do not understand in detail what influences the cells in the hypoganglionic section of the megacolon in promoting the inflammatory process, nor the impact of the ganglion cells of the euganglionic megacolon. Understanding the interaction between ganglion cells and immune cells in the case of postoperative enterocolitis seems even more difficult. Is this caused by a disturbance in the peristalsis behind it, even though the colon should be functioning after the intervention? Does residual stenosis play a role? Is sphincter achalasia a possible cause? Based on these questions, this topic warrants further exploration.

A few centuries after the initial discovery of congenital megacolon in 1691 by Frederick Ruysch, a Dutch Anatomist, and its detailed description in 1886 by Harald Hirschsprung, a Danish pediatrician, HSCR is still very challenging. It will need a comprehensive platform of tools and minds to understand in detail the etiopathogenesis, broaden diagnostic tools, and set up a non-surgical treatment. Scientists and physicians need to apply future research efforts to tackle this Sisyphean task.
